# Supplementary material for: Mechanisms of Deamidation of Asparagine Residues and Effects of Main-Chain Conformation on Activation Energy
Source: Int J Mol Sci. 2020 Sep 24;21(19):7035. doi: 10.3390/ijms21197035 (PMC7582646; doi:10.3390/ijms21197035)
Supplement: Supplementary file 1 [file ijms-21-07035-s001.pdf]

Supplementary Materials

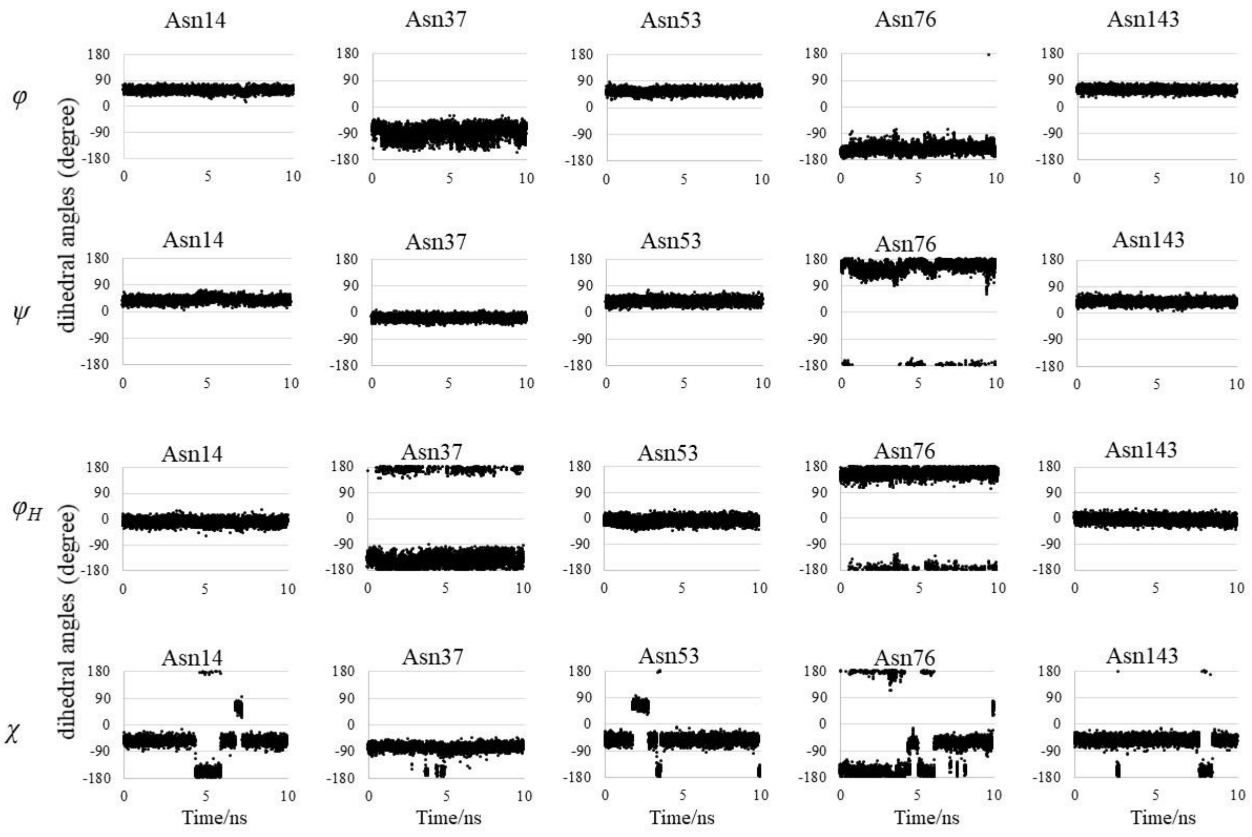

Figure S1. Dihedral angles of Asn residues in γS-crystallin for the last 100 ns simulations.

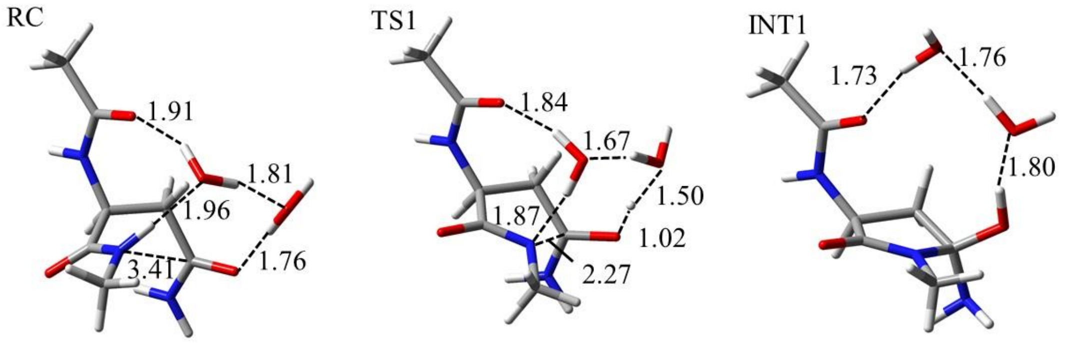

Figure S2. Optimized geometries of the cyclization step for syn conformation in a water-catalyzed reaction. Carbon, nitrogen, oxygen, and phosphorus atoms are illustrated in gray, blue, red, and orange, respectively. Selected interatomic distances are in Å.

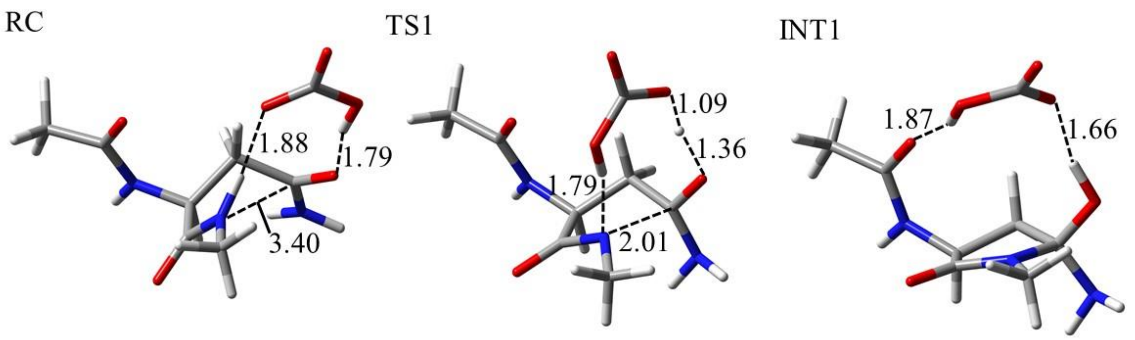

Figure S3. Optimized geometries of the cyclization step for the syn conformation in carbonate-catalyzed reaction. Carbon, nitrogen, oxygen, and phosphorus atoms are illustrated in gray, blue, red, and orange, respectively. Selected interatomic distances are in Å.

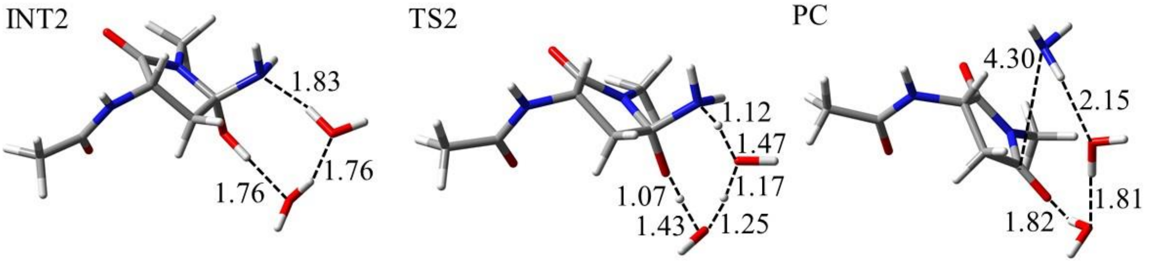

Figure S4. Optimized geometries of deammoniation step for syn conformation in water-catalyzed reaction. Carbon, nitrogen, oxygen, and phosphorus atoms are illustrated in gray, blue, red, and orange, respectively. Selected interatomic distances are in Å.

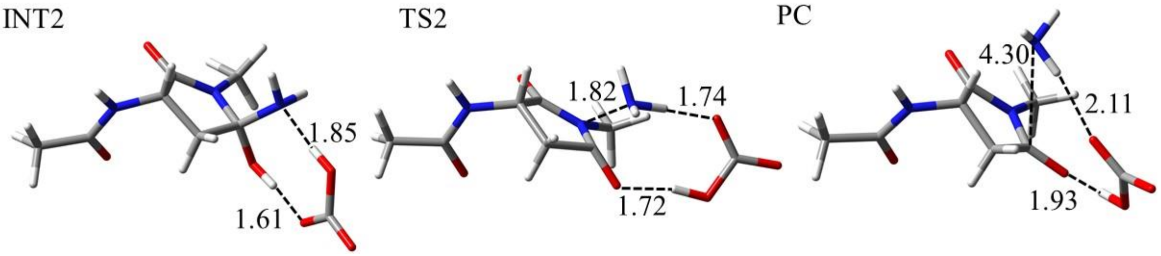

Figure S5. Optimized geometries of deammoniation step for syn conformation in carbonate-catalyzed reaction. Carbon, nitrogen, oxygen, and phosphorus atoms are illustrated in gray, blue, red, and orange, respectively. Selected interatomic distances are in Å.

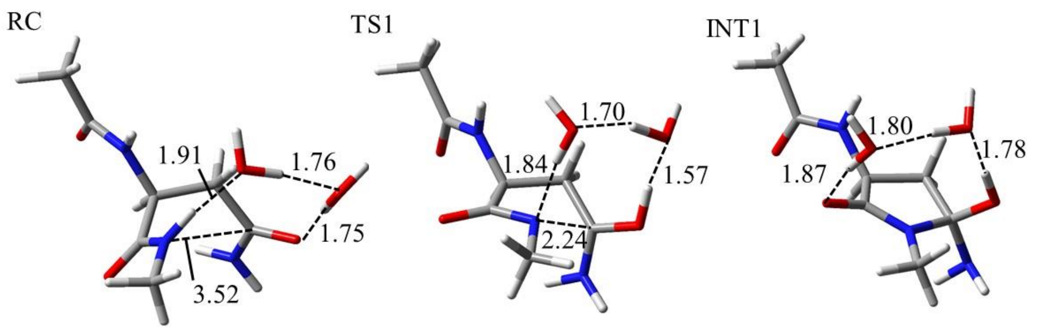

Figure S6. Optimized geometries of cyclization step for anti conformation in water-catalyzed reaction. Carbon, nitrogen, oxygen, and phosphorus atoms are illustrated in gray, blue, red, and orange, respectively. Selected interatomic distances are in Å.

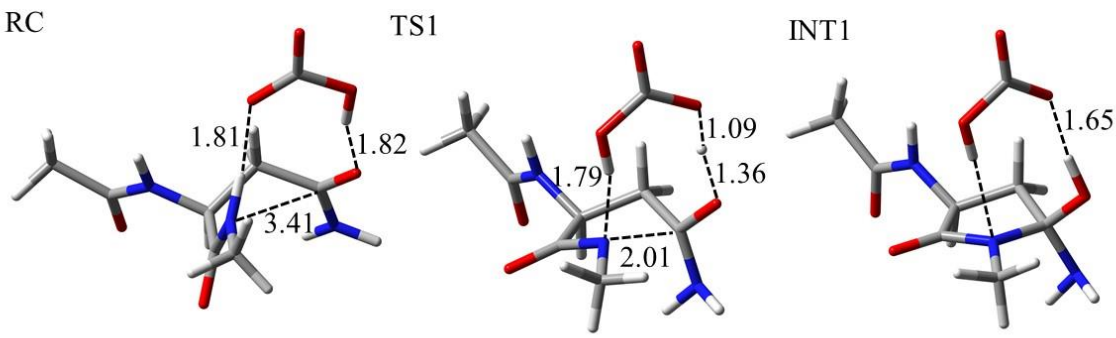

Figure S7. Optimized geometries of cyclization step for anti conformation in carbonate-catalyzed reaction. Carbon, nitrogen, oxygen, and phosphorus atoms are illustrated in gray, blue, red, and orange, respectively. Selected interatomic distances are in Å.

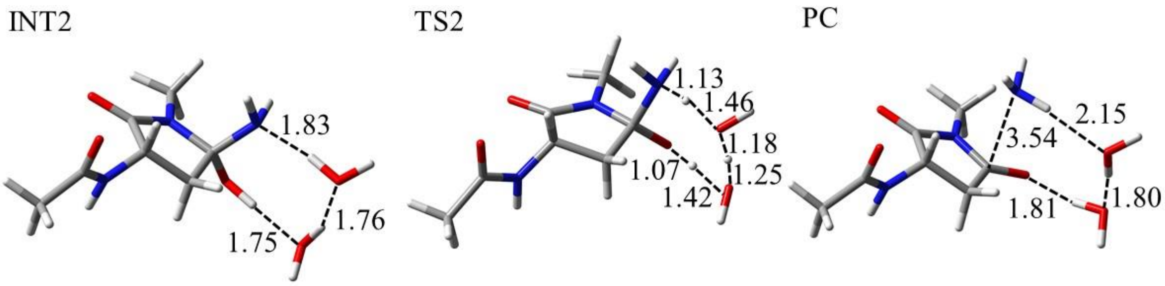

Figure S8. Optimized geometries of deammoniation step for anti conformation in water-catalyzed reaction. Carbon, nitrogen, oxygen, and phosphorus atoms are illustrated in gray, blue, red, and orange, respectively. Selected interatomic distances are in Å.

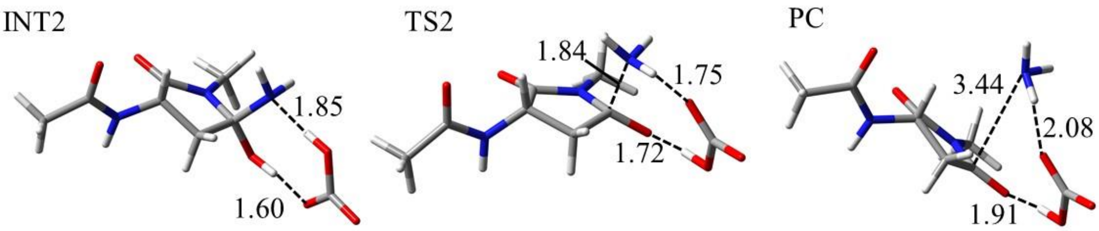

Figure S9. Optimized geometries of deammoniation step for anti conformation in carbonate-catalyzed reaction. Carbon, nitrogen, oxygen, and phosphorus atoms are illustrated in gray, blue, red, and orange, respectively. Selected interatomic distances are in Å.

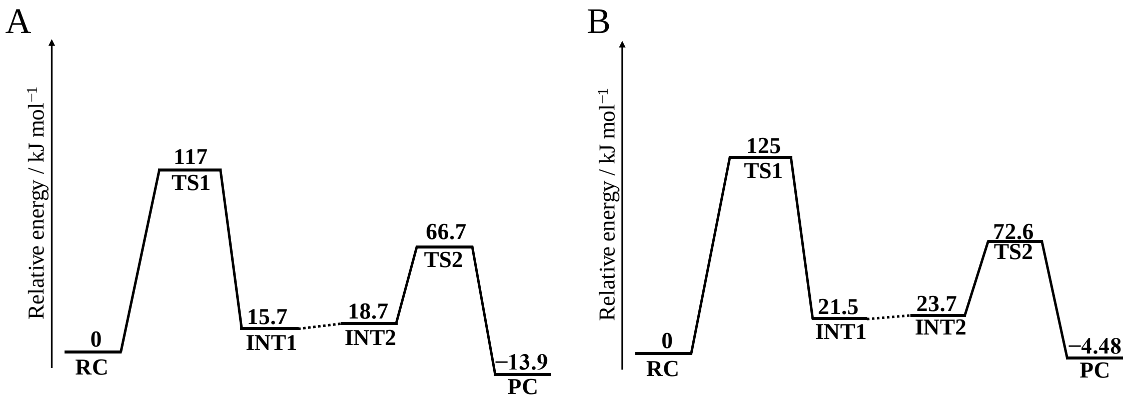

Figure S10. Relative energy profiles calculated using MP2/6-311+G(2d,2p)//B3LYP/6-31+G(d,p) level. Entire energy profiles of (A) syn and (B) anti conformation in the water-catalyzed reaction.
